# Neuronal mTORC1 is required for acute Aβ‐induced cognitive dysfunction

**DOI:** 10.1002/alz.091116

**Published:** 2025-01-03

**Authors:** Stacy A Hussong, Kylie M Williams, Veronica Galvan

**Affiliations:** ^1^ University of Oklahoma Health Sciences Center, Oklahoma City, OK USA; ^2^ Oklahoma City Veterans Health Care System, Oklahoma City, OK USA

## Abstract

**Background:**

Previous studies have found that mechanistic target of rapamycin complex 1 (mTORC1) activity is significantly increased in Alzheimer’s disease (AD) patients and mouse models of AD. Additionally, inhibition of mTORC1 with systemic rapamycin treatment ameliorates AD‐like phenotypes in several AD mouse models. However, the specific contribution of neuronal mTORC1 signaling in driving AD phenotypes has not yet been explored. In this study, we use an innovative neuron‐specific knockdown mTORC1 mouse model to determine whether neuronal mTORC1 signaling contributes to cognitive dysfunction in response to acute amyloid β (Aβ) exposure.

**Methods:**

For these experiments, we used an innovative mouse model that expresses an inducible, neuron‐specific Cre recombinase in Raptor^ff/fl^ mice. Raptor is a required component of mTORC1, thereby this Cre‐lox system results in a neuronal mTORC1 knockdown mouse. Cre expression was induced at 6 months of age. To create Aβ_1‐42_ oligomers, Aβ_1‐42_ was reconsitituted in a 10% DMSO in PBS solution (220µM) and incubated at 37°C for 48 hours. Littermate control (wildtype, Cre non‐transgenic) and neuronal mTORC1 knockdown mice were given a single intraventricular injection of vehicle (10% DMSO in PBS) or oligomerized Aβ_1‐42_ (5µL of 220µM Aβ_1‐42_) to acutely induce AD‐like cognitive deficits. Cognitive behavior was assessed by Y‐maze and contextual fear conditioning 7‐10 days after acute Aβ_1‐42_ exposure.

**Results:**

Wildtype control mice acutely exposed to Aβ_1‐42_ had significantly impaired spatial working memory (Y‐maze, p<0.01) as well as significant deficits in contextual memory (fear conditioning, p<0.05). However, we found that mice with reduced neuronal mTORC1 signaling injected with Aβ_1‐42_ did not exhibit cognitive deficits in either spatial working memory or contextual memory as compared to vehicle‐treated wildtype controls. Furthermore, we found that mice with reduced mTORC1 signaling acutely exposed to Aβ_1‐42_ had significantly improved cognitive outcomes as compared to Aβ‐injected, wildtype, control mice (Y‐maze, p<0.05; fear conditioning, p<0.05).

**Conclusion:**

Neuronal mTORC1 is required for acute Aβ‐induced cognitive dysfunction. Therefore, reduction of mTORC1 signaling in neurons with systemic rapamycin treatment contributes to the amelioration of Aβ‐induced cognitive dysfunction in AD mouse models.

